# Machine-Learned Data Structures of Lipid Marker Serum Concentrations in Multiple Sclerosis Patients Differ from Those in Healthy Subjects

**DOI:** 10.3390/ijms18061217

**Published:** 2017-06-07

**Authors:** Jörn Lötsch, Michael Thrun, Florian Lerch, Robert Brunkhorst, Susanne Schiffmann, Dominique Thomas, Irmgard Tegder, Gerd Geisslinger, Alfred Ultsch

**Affiliations:** 1 Institute of Clinical Pharmacology, Goethe-University, Theodor Stern Kai 7, Frankfurt am Main 60590, Germany; susanne.schiffmann@med.uni-frankfurt.de (S.S.); thomas@med.uni-frankfurt.de (D.T.); tegeder@em.uni-frankfurt.de (I.T.); geisslinger@em.uni-frankfurt.de (G.G.); 2 Fraunhofer Institute of Molecular Biology and Applied Ecology-Project Group Translational Medicine and Pharmacology (IME-TMP), Theodor-Stern-Kai 7, Frankfurt am Main 60590, Germany; lerch@Mathematik.Uni-Marburg.de; 3 DataBionics Research Group, University of Marburg, Hans-Meerwein-Strasse 6, Marburg 35032, Germany; mthrun@Mathematik.Uni-Marburg.de (M.T.); ultsch@Mathematik.Uni-Marburg.de (A.U.); 4 Department of Neurology, Goethe-University Hospital, Schleusenweg 2-16, Frankfurt am Main 60528, Germany; robert.brunkhorst@kgu.de

**Keywords:** bioinformatics, data science, machine-learning, multiple sclerosis, prostanoids, ceramides

## Abstract

Lipid signaling has been suggested to be a major pathophysiological mechanism of multiple sclerosis (MS). With the increasing knowledge about lipid signaling, acquired data become increasingly complex making bioinformatics necessary in lipid research. We used unsupervised machine-learning to analyze lipid marker serum concentrations, pursuing the hypothesis that for the most relevant markers the emerging data structures will coincide with the diagnosis of MS. Machine learning was implemented as emergent self-organizing feature maps (ESOM) combined with the U*-matrix visualization technique. The data space consisted of serum concentrations of three main classes of lipid markers comprising eicosanoids (*d* = 11 markers), ceramides (*d* = 10), and lyosophosphatidic acids (*d* = 6). They were analyzed in cohorts of MS patients (*n* = 102) and healthy subjects (*n* = 301). Clear data structures in the high-dimensional data space were observed in eicosanoid and ceramides serum concentrations whereas no clear structure could be found in lysophosphatidic acid concentrations. With ceramide concentrations, the structures that had emerged from unsupervised machine-learning almost completely overlapped with the known grouping of MS patients versus healthy subjects. This was only partly provided by eicosanoid serum concentrations. Thus, unsupervised machine-learning identified distinct data structures of bioactive lipid serum concentrations. These structures could be superimposed with the known grouping of MS patients versus healthy subjects, which was almost completely possible with ceramides. Therefore, based on the present analysis, ceramides are first-line candidates for further exploration as drug-gable targets or biomarkers in MS.

## 1. Introduction

Lipid signaling has been suggested to be, among others [1], a major pathophysiological mechanism of multiple sclerosis (MS) [2], up to the hypothesis that MS would be in fact a disease of lipid metabolism [3]. Among lipids, cholesterol and cholesterol turnover products have been associated with MS [4], whereas omega-3 lipids were protective by preserving the blood brain barrier [5]. Recent investigations point at several further classes of lipids that are regulated in MS. Currently, a scientific focus centers on eicosanoids including hydroxyeicosatetraenoic acids [6], ceramides, and lyosophosphatidic acids [7,8]. Along with the increasing knowledge about lipid signaling emerging from contemporary molecular research, the acquired data become increasingly complex, which is acknowledged in proposals to implement bioinformatics methods in lipid research [9,10,11].

**Figure 1:**
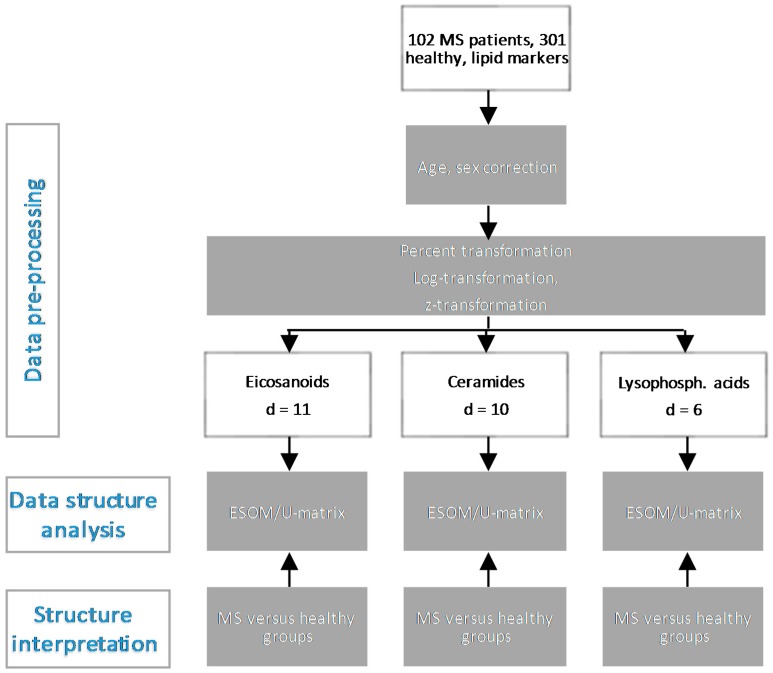
Flow chart of the data analysis. The figure provides an overview on the applied machine-learning approach, which was performed in three main steps (left column of boxes, indicated in blue letters): Firstly, data preprocessing was applied as indicated in the grey boxes. This provided lipid markers of three different classes (white rectangles) that formed the data spaces. Secondly, structure identification in the data space was performed by means of unsupervised machine-learning with the emergent self-organizing feature maps (ESOM)/U-matrix method [12]. Thirdly, the identified structures were superimposed with the known grouping of the data into multiple sclerosis (MS) patients and healthy subjects. The agreement between identified and known structure was then analyzed (structure interpretation).

In the present work, a bioinformatics approach was therefore adopted to explore the possible role of lipids in MS. Specifically, the hypothesis was pursued that lipid markers display distinct serum concentration patterns and that these patterns will be complex (Figure 1). However, the patterns were not approached mechanistically such as via analysis of classical metabolic pathways. Instead, the emergence of distinct patterns in MS was approached from a data-science perspective following the expectation that for the most relevant markers, the data structures will emerge that coincide with the grouping of subjects into MS patients or healthy controls. Serum concentrations of three main classes of lipid markers comprising eicosanoids, ceramides, and lyosophosphatidic acids were analyzed by applying unsupervised machine learning with the task to find data structures. From the agreement between the distance and density based structures and the known separation of the data into MS patients or healthy subjects (prior classification), it was expected to obtain hints at the suitable lipid marker class for future biomarker or drug development in MS.

## 2. Results and Discussion

### 2.1. Data Structures of Eicosanoid Concentrations

**Figure 2:**
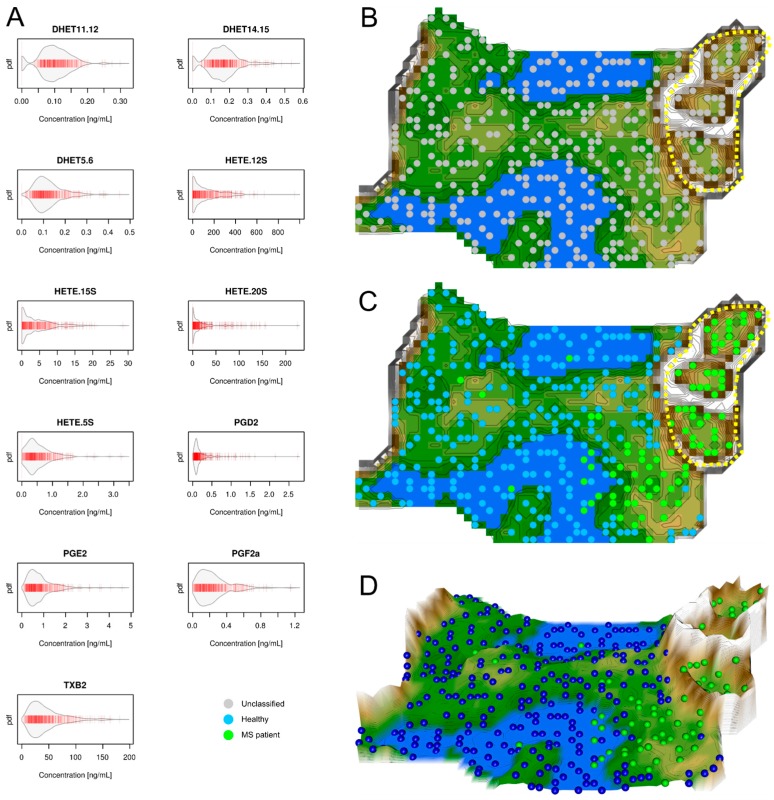
Data structures of eicosanoid serum concentrations: Left part, (**A**) Serum concentrations of *d* = 11 eicosanoids (raw data). The data are shown in alphabetical order of lipid mediator names. The beanplots [13] show the individual observations as small lines in a one-dimensional scatter plot, surrounded by a mirrored kernel density estimation of the distributions. Each panel displays a single eicosanoids marker. PGD2 = prostaglandin D2, PGE2 = prostaglandin E2, PGF2a = prostaglandin F2a, TXB2 = thromboxane, DHET = dihydroxyeicosatrienoic acid, HETE = hydroxyeicosatetraenoic acid. Right part: U*-matrix visualization of distance and density based structures of the eicosanoid serum concentration (*d* = 11 eicosanoid markers) observed in *n* = 102 multiple sclerosis patients and *n* = 301 healthy subjects. The figure has been obtained using a projection of the data points onto a toroid grid of 1440 neurons where the opposite edges are connected. The U*-matrix visualization was colored as a top view of a topographic map with brown (up to snow-covered) heights and green valleys with blue lakes. Valleys indicate clusters and watersheds indicate borderlines between different clusters. The dots indicate the so-called “best matching units” (BMUs) of the self-organizing map (SOM), which are those neurons whose weight vectors are most similar to the input. A single neuron can be the BMU for more than one data point or subject, hence, the number of BMUs may not be equal to the number of subjects as in the present case. Differently colored BMUs represent healthy versus MS patient groups; (**B**) Projection of the markers shown in A onto a self-organizing map. On the raw U*-matrix, the BMUs are colored neutrally (grey). A structure consisting of two clusters emerges. One large cluster and a separate second cluster, which is heterogonous in itself suggesting two subclusters. This region at the upper right part of the U*matrix is marked with a dotted yellow line; (**C**) Analysis of the agreement between the data set structure and grouping of the cohort. When the group membership to either the MS patients (green dots) or the healthy subjects (blue dots) is projected onto the U*-matrix, it becomes clear that the separate cluster surrounded by the yellow dotted line contains only MS patients. However, patients also are found in the first cluster (the green dots outside the yellow-surrounded region) indicating that the eicosanoids serum concentrations are insufficient to separate patients from healthy subjects; (**D**) A topographic map of the U-matrix visualization of distance and density based structures of the eicosanoid serum concentrations. It again shows that a fraction of the MS patients is located outside the yellow-surrounded cluster, which is clearly separated from the other cluster by a mountain range. The figure has been created using the R software package (version 3.3.2 for Linux; Available online: http://CRAN.R-project.org/ [14]). Specifically, the beanplots have been drawn using the R package “beanplot” (Kampstra, P.; Available online: https://cran.r-project.org/package=beanplot [13]) and the figures displaying geographical map analogies have been created using our R library “Umatrix” (M. Thrun, F. Lerch, Marburg, Germany, Available online: http://www.uni-marburg.de/fb12/arbeitsgruppen/datenbionik//software; file, Available online: http://www.uni-marburg.de/fb12/arbeitsgruppen/datenbionik//umatrix.tar.gz).

Serum concentrations of *d* = 11 eicosanoids lipid serum markers were available from 102 patients with multiple sclerosis and 301 healthy subjects (Figure 2 left). Unsupervised machine learning was applied to identify the structures in the data space $D=\left\{x_{i},i=1,\ldots ,n\right\}\subset R^{d}$, i.e., the acquired variables were respectively *d* = 11 serum eicosanoid concentrations, *x*, that comprised the *n* = 403 subjects. Following projection of the vector space onto a toroid grid of 30 × 48 = 1440 neurons and training of a self-organizing map (SOM), a U*-matrix visualization was displayed on top of this SOM (Figure 2 right). This provided an emergent self-organizing feature map (ESOM), in which large U-heights in the U*-matrix visualization indicated a large gap in the data space whereas low U-heights indicated that the points are close to each other in the data space indicating structure in the data set. On the topographic map of the U-matrix (Figure 2D), valleys, ridges, and basins enhance the visibility of the structure of clusters.

This structure seen on the U*-matrix visualization indicated two main clusters (Figure 2). A larger cluster comprised mainly healthy subjects whereas a smaller cluster emerged that comprised almost exclusively MS patients (surrounded by a yellow dotted line in Figure 2). However, a projection of the original classification into MS patients versus healthy subjects showed that the cohort was only incompletely separated by the eicosanoids serum concentration data structure. A considerable fraction of MS patients belonged to the first cluster; however, almost none of the healthy subjects belonged to the second cluster. This insufficient separation was reflected in moderate performance when applying standard performance analyses that resulted in a sensitivity and specificity of the data structure correctly reflecting the grouping of 54% and 100%, respectively, and a balanced accuracy of 77%.

### 2.2. Data Structures of Ceramide Concentrations

**Figure 3:**
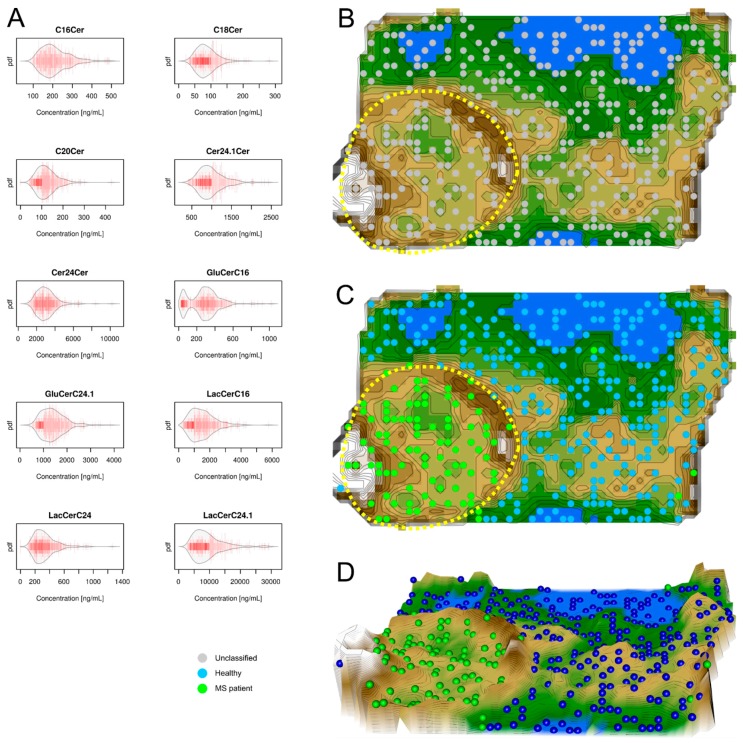
Data structures of ceramide serum concentrations: Left part, (**A**) Serum concentrations of *d* = 10 ceramides (raw data, complete cohort). The data are shown in alphabetical order of lipid mediator names. The beanplots [13] show the individual observations as small lines in a one-dimensional scatter plot, surrounded by a mirrored kernel density estimation of the distributions. Cer = ceramide, GluCer = glucosylceramide, LacCer = lactosylceramide. Right part, U*-matrix visualization of the distance and density based structures of the ceramide serum concentrations (*d* = 10 ceramide markers) observed in *n* = 102 multiple sclerosis patients and *n* = 301 healthy subjects. The figure has been obtained using a projection of the data points onto a toroid grid of 1440 neurons where the opposite edges are connected. The U*-matrix was colored as a geographical map with brown heights and green valleys with blue lakes. Valleys indicate clusters and watersheds indicate borderlines between different clusters. The dots indicate the so-called “best matching units” (BMUs) of the self-organizing map (SOM), which are those neurons whose weight vector is most similar to the input. A single neuron can be the BMU for more than one data point or subject, hence, the number of BMUs may not be equal to the number of subjects as in the present case. Differently colored BMUs represent healthy versus MS patient groups; (**B**) projection of the markers shown in A onto a self-organizing map. On the raw U-matrix, the BMUs are colored neutrally. A cluster structure emerges as two separate clusters. One cluster region at the lower left part of the U*matrix is marked with a dotted yellow line; (**C**) analysis of agreement between the data structure and grouping of the cohort. When the group membership to either the MS patients (green dots) or the healthy subjects (blue dots) is projected onto the U*-matrix, it becomes clear that the separate cluster surrounded by the yellow dotted line contains only MS patients while the remaining cluster contains nearly only healthy subjects. This indicates an almost perfect separation of patients from healthy subjects by the ceramide serum concentrations (except a few outliers, i.e., green dots among the blue-dots zone); (**D**) a topographic map of the U-matrix visualization of distance and density based structures of the ceramide serum concentrations. The figure has been created using the R software package (version 3.3.2 for Linux; Available online: http://CRAN.R-project.org/ [14]). Specifically, the beanplots have been drawn using the R package “beanplot” (Kampstra P.; Available online: https://cran.r-project.org/package=beanplot [13]) and the figures displaying geographical map analogies have been created using our R library “Umatrix” (M. Thrun, F. Lerch, Marburg, Germany, Available online: http://www.uni-marburg.de/fb12/arbeitsgruppen/datenbionik//software; file, Available online: http://www.uni-marburg.de/fb12/arbeitsgruppen/datenbionik//umatrix.tar.gz).

Unsupervised machine learning was applied to identify structures in the data space $D=\left\{x_{i},i=1,\ldots ,n\right\}\subset R^{d}\;$, of *d* = 10 serum ceramide concentrations (Figure 3 left), *x_i_*, acquired in the *n* = 403 subjects. Following the projection of the vector space onto a toroid grid of 30 × 48 = 1440 neurons and training of a self-organizing map, a U*-matrix visualization was displayed on top of this SOM (Figure 3 right). The cluster structure seen on the U*-matrix visualization suggested two main clusters (Figure 3). A large cluster comprised almost exclusively healthy subjects whereas a smaller cluster emerged that comprised almost exclusively MS patients (surrounded by a yellow dotted line in Figure 3). Compared to the eicosanoid grouping (prior classification), a projection of the original classification into MS patients versus healthy subjects onto the ceramide distance and density based data structures showed that the cohort was almost completely separated by the ceramide serum concentration data structure. This was reflected in a comparatively better performance of the ceramide-based U*-matrix visualization when applying standard test analyses that resulted in a sensitivity and specificity of 89.2% and 100%, respectively, and a balanced accuracy of 94.6%.

### 2.3. Data Structures of Lysophosphatidic Acid Concentrations

**Figure 4:**
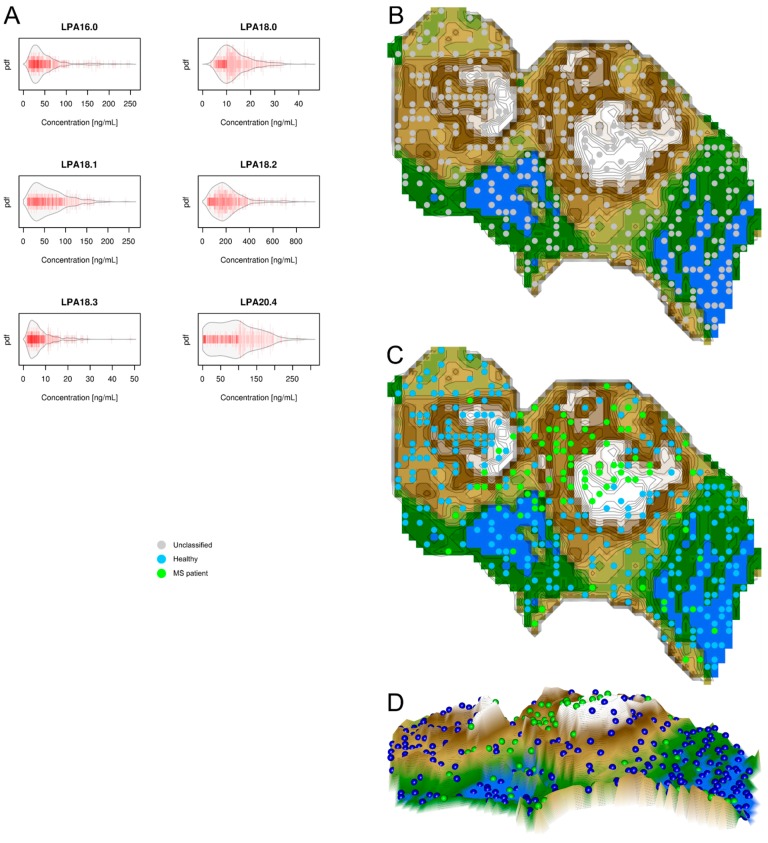
Data structures of lysophosphatidic acid serum concentrations: Left part, (**A**) Serum concentrations of *d* = 6 lysophosphatidic acids (raw data, complete cohort). The data are shown in alphabetical order of lipid mediator names. The beanplots [13] show the individual observations as small lines in a one-dimensional scatter plot, surrounded by a mirrored kernel density estimation of the distributions. Each panel displays a single lysophosphatidic acid marker. LPA = lysophosphatidic acid. Right part: U*-matrix visualization of distance and density based structures of the lysophosphatidic acid serum concentrations (*d* = 6 lysophosphatidic acid markers) observed in *n* = 102 multiple sclerosis patients and *n* = 301 healthy subjects. The figure has been obtained using a projection of the data points onto a toroid grid of 1440 neurons where the opposite edges are connected. The U*-Matrix was colored as a geographical map with brown (up to snow-covered) heights and green valleys with blue lakes. Valleys indicate clusters and watersheds indicate borderlines between different clusters. The dots indicate the so-called “best matching units” (BMUs) of the self-organizing map (SOM), which are those neurons whose weight vectors are most similar to the input. A single neuron can be the BMU for more than one data point or subject, hence, the number of BMUs may not be equal to the number of subjects as in the present case. Differently colored BMUs represent healthy versus MS patient groups; (**B**) Projection of the markers shown in A onto a self-organizing map. On the raw U-matrix, the BMUs are colored neutrally and no real cluster structure emerges; (**C**) Analysis of agreement between the marker structure and grouping of the cohort. When the group membership to either the MS patients (green dots) or the healthy subjects (blue dots) is projected onto the U*-matrix, it becomes clear that it does not coincide with the data set’s structure displayed as the U-matrix; (**D**) A topographic map of the U-matrix visualization of distance and density based structures of the lysophosphatidic serum concentrations. It shows the comparatively weak structure found in the lysophosphatidic acid serum concentrations. No clear ridges-surrounded valleys can be seen; the map mainly consists of “mountains”, which does not allow for concluding a valid cluster structure in the data. Specifically, the beanplots have been drawn using the R package “beanplot” (Kampstra P.; Available online: https://cran.r-project.org/package=beanplot [13]) and the figures displaying geographical map analogies have been created using our R library “Umatrix” (M. Thrun, F. Lerch, Marburg, Germany, Available online: http://www.uni-marburg.de/fb12/arbeitsgruppen/datenbionik//software; file, Available online: http://www.uni-marburg.de/fb12/arbeitsgruppen/datenbionik//umatrix.tar.gz).

Serum concentrations of *d* = 6 lysophosphatidic acids serum markers are shown in Figure 4 to the left. Unsupervised machine learning was applied to identify the structures in the data space $D=\left\{x_{i},i=1,\ldots ,403\right\}\subset R^{d}\;$, i.e., the acquired variables respectively *d* = 6 serum lysophosphatidic acid concentrations, *x*, that comprised the *n* = 403 subjects. Following the projection of the data space onto a toroid grid of 30 × 48 = 1440 neurons and training of a self-organizing map, a U*-matrix visualization was displayed on top of this SOM (Figure 4 right). Although the preprocessing of the data was identical to the two cases above, a cluster structure could not be identified in the visualization of the U*-matrix. On the U-matrix visualization, scattered, separate mountains emerged, but no clear valleys surrounded by separating mountain ridges, which does not allow for concluding a major cluster structure in the data. Consistent with the absence of structures, a projection of the original groups onto the SOM showed no obvious overlap with the data structure.

Results of the present analysis support that lipid marker serum concentrations form distance and density based data structures that allow for the separation of MS patients from healthy subjects in the case of distinct ceramide concentrations. This outcome is based on the recognition of structures in the data of serum concentrations of different classes of lipids using unsupervised machine-learning. The presently applied ESOM [15] method employs a structure-preserving projection of high-dimensional data points onto a two-dimensional self-organizing network while the U-matrix [16] allows for visual (in-) validation of cluster structures in the data. This has been shown to identify natural cluster structures in artificial [16] and biomedical data sets [12]. The method outperforms classical clustering methods that occasionally impose structures on data that are clearly devoid of any data structures [12]. Therefore, the cluster structures observed in the present data sets can be considered to reflect natural clusters [17]. This allows drawing two major topical conclusions from the present analysis. Firstly, the results provide support that MS is associated with altered lipid signaling, and secondly, the involvement of different classes of lipids in MS is unequal, pointing at particularly promising research directions.

The first result of the present unsupervised machine-learned data analysis supports a regulation of lipids in MS. This is biologically highly plausible and agrees with descriptions made more than 30 years ago [2]. It is compatible with the increasing recognition of the roles of bioactive lipids in modulating immune response and neuronal functions [18]. The earliest investigations pointed at arachidonic acid derivatives, in the presence of increased phospholipase activity, as a class of lipids being regulated in MS [2]. A regulation of arachidonic acid derivatives including prostaglandins and hydroxyeicosatetraenoic acids has been verified in comparisons of blood concentrations measured in MS patients or in healthy subjects [6], and in particular anti-inflammatory arachidonic acid-based lipid mediators have been repeatedly addressed in the context of MS [19]. A pathophysiological relationship between lipid metabolism and MS has been proposed in the present decade by relating the metabolism of cholesterol with MS, which has been suggested to be triggered by toxic derivatives of low-density lipoproteins via altered activity of peroxisome proliferator-activated receptors leading to changes in the cholesterol metabolism, in addition to immune system changes [3,20]. In line with this research, cholesterol and its turnover products have been proposed as biomarkers for MS [4]. Further classes of lipid mediators regulated in MS include sphingolipids [21], ceramides, or lysophosphatidic acids [7,8].

The second result of the present machine-learned analysis indicates a different importance of lipid mediator classes for MS. Specifically, the agreement of the identified distance and density based data structures in lipid mediator serum concentrations with the grouping of the present cohort comprising MS patients or healthy subjects was nearly complete with ceramides. By contrast, with eicosanoids the structures and grouping only partly overlapped and grouping was not possible with lysophosphatidic acids. This indicates that eicosanoids seem to be regulated in MS, however, this regulation probably overlaps with several other reasons of eicosanoid regulation to which MS patients are exposed to [22]. This interpretation accommodates the observation in the U-matrix (Figure 2) that a cluster was almost exclusively populated with MS patients, indicating a specific regulation of eicosanoids; however, MS patients were also found in the other cluster among healthy subjects, indicating unspecific changes in prostanoid concentrations that are similar in MS patients and healthy subjects. The different degrees of involvement agrees with the previous reports of, for example, only modest effects of the serum lipid profile on disease progression in MS when analyzing low and high density lipoproteins, total cholesterol, and triglycerides [20]. The results of the present analysis, which had a focus on identifying the structure in the data that agreed with the known data grouping, therefore point at the ceramide system as providing possible biomarkers or drug targets for MS.

The advice of using ceramides as candidate biomarkers or to initiate research on druggable targets into this direction, however, cannot be entirely based on the analysis of the present data set due to limitations of the present data set. The present separation of the cohort based on structure identification in the data was obtained in a data set comprising only either MS patients or healthy subjects. Whether the same separation with ceramides would persist when patients with differential disease activity of MS, or diagnoses of other inflammatory CNS diseases, brain tumors, spinal ischemia, sarcoidosis, vasculitis, acute disseminated encephalopathy, or leukodystrophy, are included could not be addressed. Moreover, the present structure was obtained from data from a cohort with an established MS diagnosis. Whether a ceramide-based biomarker would detect MS in its early stages or predict if minimal disease would progress to MS and a biomarker would be needed most [23,24] cannot be concluded from the present data.

## 3. Methods

### 3.1. Data Acquisition and Lipid Serum Concentration Analytics

Blood sampling and data acquisition were in agreement with the Declaration of Helsinki and were approved by the Ethics Committee of the Medical Faculty of the Goethe-University, Frankfurt am Main, Germany (protocol numbers #110/10, 27 July 2010 and #197/13, 17 June 2017). Informed written consent was obtained from all subjects. The study cohort included *n* = 102 patients with a neurologically verified diagnosis of multiple sclerosis (aged 18.2–62.8 years, 31 men). For comparison, samples available from *n* = 301 healthy subjects (aged 18–53.2 years, 118 men) were included. Inclusion criteria had been age ≥18 years, absence of current medical conditions queried by medical interview, no drug intake for at least one week excluding contraceptives, vitamins, and hormone substituting drugs, and no excessive body weight (BMI 22.2, interquartile range: 20.3–24.1, compare [25]).

Venous blood samples (9 mL) were collected into serum tubes and centrifuged at 3000 rpm for 10 min. Serum was separated and frozen at −80 °C until assay. Serum concentration analytics were performed using liquid chromatography-electrospray ionization-tandem mass spectrometry (LC-ESI-MS/MS) essentially as described previously [26,27]. In brief, eicosanoids (DHET11.12, DHET14.15, DHET5.6, HETE.12S, HETE.15S, HETE.20S, HETE.5S, PGD2, PGE2, PGF2a, TXB2; PGD2 = prostaglandin D2, PGE2 = prostaglandin E2, PGF2a = prostaglandin F2a, TXB2 = thromboxane, DHET = dihydroxyeicosatrienoic acid, HETE = hydroxyeicosatetraenoic acid) were assayed in two different LC-MS/MS runs. Prostanoids were separated on a Synergi Hydro column (150 × 2 mm, 4 µm, Phenomenex) using water with 0.0025% formic acid and acetonitrile with 0.0025 formic acid as mobile phases. The analysis of HETE/DHET was done with a Gemini NX column (150 × 2 mm, 5 µm, Phenomenex) using water with 0.01% ammonia and acetonitrile with 0.01% as mobile phases. In both cases, quantification was performed using a triple quadrupole mass spectrometer QTRAP 5500 (Sciex, Darmstadt, Germany) equipped with a Turbo-V-source operating in negative ESI mode. Ceramides (C16Cer, C18Cer, C20Cer, Cer24Cer, Cer24.1Cer, GluCerC16, GluCerC24.1, LacCerC16, LacCerC24, LacCerC24.1; Cer = ceramide, GluCer = glucosylceramide, LacCer = lactosylceramide) were analyzed using a Luna C18 column (150 × 2 mm ID, 5 µm particle size, Phenomenex) coupled to an API 4000 mass spectrometer equipped with an APCI (Atmospheric Pressure Chemical Ionization) ion source operating in positive mode (Sciex) for ceramides and equipped with an ESI source for GluCer and LacCer. In both cases, the mobile phases were water with 0.1% formic acid and acetonitrile/tetrahydrofuran/formic acid (49.95:49.95:0.1; *v/v/v*). The analysis of lysophosphatidic acids (LPA16.0, LPA18.0, LPA18.1, LPA18.2, LPA18.3, LPA20.4) was performed using a Mercury Luna C18 column (20 × 2 mm, 3 µm, Phenomenex, Aschaffenburg, Germany) coupled to a triple quadrupole mass spectrometer (QTRAP 5500, AB Sciex Germany GmbH, Darmstadt, Germany) operating in negative ESI mode. Mobile phases were water with 50 mM ammonium formate and 0.2% formic acid and acetonitrile/isopronanol/formic acid (49.9:49:9:0.2; *v/v/v*). In all cases, the analytes were extracted using liquid-liquid-extraction prior to the LC-MS/MS analysis. Sample volumes were 200 µL each for prostanoids and DHET/HETE, 20 µL for ceramides, and 50 µL for LPA. For all analytes, the concentrations of the calibration standards, quality controls, and samples were evaluated by Analyst software 1.6 and MultiQuant Software 3.0 (Sciex) using the internal standard method (isotope-dilution mass spectrometry). Calibration curves were calculated by linear regression with 1/x weighting for ceramides and LPA and by quadratic regression with 1/x^2^ weighting for eicosanoids.

### 3.2. Data Analysis

Data were analyzed using the R software package (version 3.3.2 for Linux; Available online: http://CRAN.R-project.org/ [14]) on an Intel Xeon^®^ computer running on Ubuntu Linux 16.04.1 64-bit. An overview of the analytical steps is shown in Figure 1. The data space consisted of *d* = 11 eicosanoid markers, or of *d* = 10 ceramide markers, or of *d* = 6 lysophosphatidic acids markers, measured in the serum from MS patients or healthy subjects. Unsupervised machine learning was used to analyze the data space $D=\left\{x_{i},i=1,\ldots ,n\right\}\subset R^{d}$ comprising lipid marker concentrations with the task of finding the distance and density based structures in the data. This was addressed by means of emergent self-organizing feature maps (ESOM) [15] combined with the U-matrix [16] and its visualization technique as a recently shown unbiased method to identify structures in biomedical data [12]. Subsequently, the identified structure in the data, if any, was explored with respect to the degree, at which it resembled the known grouping of MS patients versus healthy subjects (Figure 1).

The exploration of the data space was preceded by data preprocessing (Figure 1). An outlier in the DHET5.6 serum concentrations was eliminated on the basis of a significant Grubbs test [28] (G = 19.624, U = 0.03966, *p* < 2.2 × 10^−16^). Influences of age or sex were reduced by applying corrections based on linear regression or median differences, respectively. To obtain a uniform scaling of all marker serum concentrations suitable to be assessed for Euclidean distances, data were transformed into percentages [29], i.e., into the interval [0,100]. As quantile-quantile plots pointed at log-normal distribution of the data, which is in line with general observations in blood-derived concentration data [30], the data was zero invariant log-transformed and subsequently, z-transformation was applied.

The data, respectively data space $D=\left\{x_{i},i=1,\ldots ,n\right\}\subset R^{d}\;$, *n* = 403, where *x_i_* comprised the serum concentrations of lipid markers, was explored for emergent group/cluster structures using unsupervised machine learning [31]. The aim of this analysis was to find interesting structures in the data space accessible to subsequent interpretation with respect to the MS patient distribution. It should be remarked that the information about the presence or absence of the disease was not included in this analysis (unsupervised), which exclusively explored the data spaces composed of either eicosanoid, ceramide, or lysophosphatidic acid serum concentrations. The grouping, i.e., MS patients versus healthy subjects (prior classification), was only regarded at the final steps after a structure in the data had been found (see below).

Unsupervised machine-learning was performed by employing a self-organizing artificial neuronal network of the Kohonen type (emergent self-organizing map, ESOM) [12,32,33]. The neural network consisted of a two-dimensional toroid grid [16] of neurons with 30 rows and 48 columns (u = 1440 units). Each neuron *i* held, in addition to a position on the two-dimensional grid $m_{i},$ a corresponding set of weights (prototype)$w\left(m_{i}\right)$, which is of the same dimension as the high-dimensional space of the preprocessed lipid marker concentrations. These weights were initially randomly drawn from the range of the data variables and subsequently adapted to the data during the learning phase that used 20 epochs, i.e., sweeps through the data.

The trained ESOM presented the subjects on a two-dimensional toroid map as the localizations of “best matching units” (BMU), which are special prototypes that are assigned unambiguously to data space points (here subjects). On top of this grid, the distances between data points are calculated with the U-matrix [32,33]. Every value (height) in the U-matrix depicts the average high-dimensional distance of a prototype to all immediate neighboring prototypes regarding a grid position. The U-matrix can be enhanced by calculating a P-matrix [16] displaying the point density $p\left(m_{i}\right)=\left|\left\{data\;points\;x_{i}\in D|d\left(x_{i},w\left(m_{i}\right)\right)\langle r\rangle 0,r\in R\right\}\right|$ estimated as the number of data points in a sphere with a radius *r* around *x* at each grid point on the ESOM’s output grid. The U*-matrix combines distance structures (U-matrix) and density structures (P-matrix) into a single matrix [16]. The corresponding visualization technique is a topographical map with hypsometric colors [34] facilitating the recognition of distance and density based structures. On this visualization technique, large “heights” in brown and white colors represent large distances between data points (here subjects) separating “valleys” in green and blue colors that represent data points that are similar. Finally, the points lying in the data structures shown in the visualization that had emerged from unsupervised machine-learning analysis were superimposed with the known grouping of MS patients versus healthy subjects (prior classification) and the agreement between the data structures with the grouping of MS patients versus healthy subjects was explored by applying standard measures of diagnostic test performance [35,36].

## 4. Conclusions

In the present report, unsupervised machine-learned data analysis [31] was applied to data sets comprising eicosanoid, ceramide, or lysophosphatidic acid lipid marker serum concentrations assayed in the blood serum of MS patients. Biochemical mechanistic bases of this observation were not addressed in this bioinformatical analysis that took a “birds eye” perspective on complex lipid related data. It was demonstrated that the combination of contemporary data science with analytical techniques for biomarkers allows, via recognition of the structure in the data, the separation of MS patients from healthy subjects based on lipid marker serum concentration data structures. This was not equally possible with any class of the three classes of lipid markers analyzed in the present work. Based on serum concentrations, the lipid-marker class of ceramides allowed an almost complete separation of MS patients from healthy subjects, whereas eicosanoids provided only a partial separation and lysophosphatidic acids lacked a consistent data structure in the present cohort. Therefore, the present unsupervised machine-learned data analysis applied on serum concentrations may complement mechanistic biochemical research by pointing out ceramides as first-line candidate lipids for further exploration in a MS context.
